# Does dietary inulin affect biological activity of a grapefruit flavonoid-rich extract?

**DOI:** 10.1186/1743-7075-9-31

**Published:** 2012-04-11

**Authors:** Adam Jurgoński, Jerzy Juśkiewicz, Karolina Kowalska, Zenon Zduńczyk

**Affiliations:** 1 Division of Food Science, Institute of Animal Reproduction and Food Research, Polish Academy of Sciences, Tuwima 10, 10-748 Olsztyn, Poland

**Keywords:** Grapefruit flavonoids, Inulin, Large intestine, Blood lipid profile

## Abstract

**Background:**

The aim of the study was to verify that the concomitant presence of grapefruit flavonoid extract with inulin in a Western-type diet may provide synergistic effects to the hindgut metabolism, as well as blood lipid and mineral profiles.

**Methods:**

Forty male Wistar rats were distributed into 4 groups and fed for 28 days with diets rich in fat, cholesterol and protein. A two-way repeated measures ANOVA was applied to assess the effects of inulin (v. sucrose, 5% of the diet), the addition of dietary grapefruit flavonoid extract (diets without or with 0.3% of an extract from hard parts of grapefruit) and the interaction between these two dietary factors.

**Results:**

When compared to the control sucrose-containing diet, the diet enriched with inulin led to typical changes within the caecum, the main part of hindgut fermentation in rats, such as acidification of the digesta, support of bifidobacteria growth and increase of propionate and butyrate production. The dietary grapefruit flavonoid extract without inulin increased the bulk and pH value of caecal digesta, whereas short-chain fatty acid concentration and the bifidobacteria population were lowered compared to the extract-free diets. Simultaneous dietary addition of both tested components decreased slightly the pH value and increased somewhat the bifidobacteria number and the propionate concentration, however to the level observed with the control sucrose-containing diet. With regard to blood lipids, dietary grapefruit flavonoid extract decreased the triglyceride concentration regardless of the dietary carbohydrate type.

**Conclusion:**

Inulin does not provide any additional benefit to the blood lipid profile caused by the dietary application of grapefruit flavonoid extract and it does not counteract clearly detrimental effects of the extract in the hindgut. Adding grapefruit extract to the diet must be performed with caution due to possible adverse hindgut responses with overdoses.

## Background

In economically developed countries, a high intake of processed, energy-dense foods, such as French fries, processed red meats, high-fat dairy products and refined grains and sweets, that contain high amounts of saturated fat, cholesterol and protein or easily absorbable carbohydrates is often called a Western diet. This dietary pattern is thought to be an important factor for the development of many diet-related diseases, such as type 2 diabetes, cardiovascular disease (CVD) or cancers [1,2]. Saturated fat and cholesterol induce dyslipidaemia and thus contribute to CVD development, whereas chronic, excessive ingestion of easily absorbable sugars that affect postprandial glycaemia and insulinaemia seem to underlay type 2 diabetes [3]. There is also a growing body of evidence that the Western diet may be a risk factor for osteoporosis through the induction of chronic, low-grade metabolic acidosis leading to the dissolution of bone minerals [4]. However, a diet abundant in whole-grain cereals, vegetables and fruits is associated with a decreased risk of diet-related diseases [5]. Notably, these health-promoting effects are due not to a single dietary constituent but rather to many factors working together, such as phytochemicals or fibre-polyphenolic complexes.

Inulin is a group of non-digestible polysaccharides occurring in many plants that are not hydrolysed by pancreatic enzymes but are broken down by specific bacteria in the hindgut, to help the large bowel function and improve the health of the organism. Among others, inulin consumption leads to the acidification of digesta and an enhancement of short-chain fatty acid (SCFA) production in the hindgut, especially propionate and butyrate. After absorption, propionate is thought to inhibit hepatic lipogenesis and positively influence the blood lipid profile [6], whereas the decreased digesta pH is probably responsible for the stimulation of mineral absorption and bone health in laboratory animals [7].

Grapefruit flavonoids are a part of the flavanone subclass, to which a number of positive and negative biological activities have been attributed. For example, flavanones are partly responsible for the interaction of grapefruit with some drugs [8]. Interestingly, some similar systemic effects following the ingestion of flavanones and inulin can occur. It has been reported that naringenin and hesperetin diglycosides (naringin and hesperidin, respectively), the main grapefruit flavonoids, significantly hindered an increase in the plasma lipids of rats fed a cholesterol-containing diet [9]. Furthermore, it has been shown that both naringin and hesperidin can improve mineral metabolism and bone health, but the exact mechanism of action remains unknown [10-13]. Interestingly, the bioavailability of flavonoids, including some grapefruit flavanones, is rather low, and the majority of these flavonoids reach the hindgut where they interact with local microbiota in a similar manner as inulin. As a result, low-molecular weight metabolites of flavanones are formed, which may contribute to their bioactivity [14].

Recent studies performed in our laboratory suggest that dietary inulin-type fructans and polyphenols can benefit the health status of the host when applied together. For example, our preliminary study showed that grapefruit flavonoids in a standard rodent diet had a negative influence on the caecum, the main part of the hindgut fermentation in rats, whereas inulin can decrease that detrimental effect [15]. Additionally, we have shown that the concomitant dietary presence of apple polyphenols and fructo-oligosaccharides increased the susceptibility of quercetin glycosides to microbial metabolism in the caecum [16]. In the present study, we verified that the concomitant presence of a grapefruit flavonoid-rich extract and inulin in a rat's Western-type diet may provide synergistic effects to caecal metabolism, as well as blood lipid and mineral profiles. A modified diet with a relatively high protein, fat and cholesterol content was used to obtain an animal model of metabolic changes related to the Westernisation of human eating habits.

## Materials and methods

### Preparations

A long-chain, sugar-free inulin (Frutafit^® ^TEX) was purchased from Sensus (Roosendaal, The Netherlands). Approximately 98% of the inulin preparation contains fructan molecules with a degree of polymerisation above 10. A commercial grapefruit extract (Cintamani, Poland) made from the stone, peel, and pith was also used in the study. The preparation consisted of flavonoids (52.8%), silicon dioxide (25%), glycerol (17.8%) and vitamin C (4.4%). The main flavonoids were diglycosides and glycosides of naringenin and hesperetin. The flavonoid composition of the preparation was characterised using a reversed-phase HPLC method (Waters system equipped with photodiode-array detector, Waters Co., Milford, MA, USA, equipped with Nova Pak C18 column, 30 cm × 3.9 mm with 4-μm packing). Two mobile phases were employed for elution: water acetic acid (98:2, v/v; A) and water acetonitrile acetic acid (78:20:2, v/v; B). The gradient profiles were 0-55 min and 100-20% for phase A and 55-70 min and 20-10% for phase B. More details regarding the flavonoid composition and method applied were given previously [15].

### Animals and diets

The local Institutional Animal Care and Use Committee approved the animal protocol used in the present study. The experiment was conducted on 40 young (4 weeks old) male Wistar rats with an average initial body weight of 81.4 ± 0.5 g that were distributed into 4 groups of ten rats each. The animals were fed for 4 weeks with an experimental diet that was a modified, standard, semi-purified casein diet for laboratory rodents recommended by the American Institute of Nutrition (Table 1). To mimic the human eating habits of economically developed countries, each diet had a high and equal content of fat, cholesterol and protein (14%, 1% and 23% of the diet, respectively). Half of the dietary fat and protein was derived from soybean oil and casein, respectively, and the rest was supplemented with pork lard and soy protein isolate, respectively. Two groups of rats were fed control (C) diets that, apart from the aforementioned, contained 5% of sucrose, and the other two groups were fed inulin (IN) diets in which 5% of the IN preparation was added instead of sucrose. Additionally, one C and IN diet was supplemented with 0.3% of the grapefruit flavonoid extract (+GFE) added to maize starch. The animals were housed in individual cages under the following standard conditions: temperature 21-22°C, relative humidity 50-70%, intensive room ventilation (15×/h), 12 h light/dark cycle, permanent access to the diets and water.

**Table 1:** Composition of the control (C) and inulin (IN) diets without or with the addition of grapefruit flavonoid extract (GFE)

Ingredient (%)	Diet			
	
	C	IN	C + GFE	IN + GFE
Casein	11.5	11.5	11.5	11.5

Soy protein isolate	11.5	11.5	11.5	11.5

DL-methionine	0.2	0.2	0.2	0.2

Sucrose	5	-	5	-

Inulin^1^	-	5	-	5

Cellulose	5	5	5	5

Maize starch	46.1	46.1	45.8	45.8

Grapefruit extract^2^	-	-	0.3	0.3

Soybean oil	7	7	7	7

Pork fat	7	7	7	7

Cholesterol	1	1	1	1

Choline chloride	0.2	0.2	0.2	0.2

Mineral mix^3^	3.5	3.5	3.5	3.5

Vitamin mix^4^	2	2	2	2

^1 ^A commercial preparation consisted of long-chain, linear-type fructans (98%) with a degree of polymerisation > 10 (Sensus, the Netherlands).

^2 ^A commercial preparation (Cintamani, Poland) from the hard parts of grapefruit (stone, peel, pith) containing flavonoids (52.8%), silicon dioxide (25%), glycerol (17.8%) and vitamin C (4.4%).

^3 ^Composition per kg mix: 357 g calcium carbonate anhydrous (40.04% Ca), 196 g potassium phosphate monobasic (22.76% P, 28.73% K), 70.78 g potassium citrate, tripotassium monohydrate (36.16% K), 74 g sodium chloride (39.34% Na, 60.66% Cl), 46.6 g potassium sulfate (44.87% K, 18.39% S), 24 g magnesium oxide (60.32% Mg), 6.06 g ferric citrate (16.5% Fe), 1.65 g zinc carbonate (52.14% Zn), 1.45 g sodium metasilicated 9H2O (9.88% Si), 0.63 g manganous carbonate (47.79% Mn), 0.3 g cupric carbonate (57.47% Cu), 0.275 g chromium potassium sulfated 12H2O (10.42% Cr), 81.5 mg boric acid (17.5% B), 63.5 mg sodium fluoride (45.24% F), 31.8 mg nickel carbonate (45% Ni), 17.4 mg lithium chloride (16.38% Li), 10.25 mg sodium selenate anhydrous (41.79% Se), 10 mg potassium iodate (59.3% I), 7.95 mg ammonium paramolybdated 4H2O (54.34% Mo), 6.6 mg ammonium vanadate (43.55% V), 221.026 g powdered sucrose.

^4 ^Composition (g/kg mix): 3.0 nicotinic acid, 1.6 Ca pantothenate, 0.7 pyridoxine-HCl, 0.6 thiamin-HCl, 0.6 riboflavin, 0.2 folic acid, 0.02 biotin, 2.5 vitamin B-12 (cyanocobalamin, 0.1% in mannitol), 15.0 vitamin E (all-rac-a-tocopheryl acetate, 500 IU/g), 0.8 vitamin A (all-trans-retinyl palmitate, 500 IU/g), 0.25 vitamin D-3 (cholecalciferol, 400 IU/g), 0.075 vitamin K-1 (phylloquinone), 974.655 powdered sucrose.

### Sample collection and analysis

Upon termination of the experiment, the rats were weighed and anaesthetised with sodium pentobarbital. Blood samples were taken from the caudal vein, and subsequently the liver, kidneys and caecum were removed and weighed. The blood was allowed to clot over 1.5 h at 37°C, and the serum was collected after centrifugation. Fresh caecal digesta was used to determine the pH, dry matter, ammonia concentration and microbiota, whereas the determination of enzyme activity and SCFA concentration were performed after storing the samples at -70°C.

The pH of the caecal digesta was measured using a microelectrode and a pH/ION meter (model 301, Hanna Instruments), and dry matter was determined at 105°C. Ammonia was extracted and trapped in a solution of boric acid then determined by a direct titration with sulphuric acid [17]. The caecal microbiota was assessed by measuring the number of bacteria in chosen populations of probiotic and potentially pathogenic microorganisms. All bacteria determinations were done immediately after sampling. Fresh caecal digesta was collected directly from each rat, weighed and immediately homogenised with 1% peptone water as a diluter. The populations of bacteria were enumerated after their cultivation on selective culture media and/or in specific incubation conditions. Bifidobacteria count was determined on Garche's agar after undergoing incubation at 37°C for 48 h in anaerobic conditions (anaerostat + AnaeroGen, Oxoid) [18]. Their identification was based on appearance of colonies, specific morphology of cells checked under phase contrast with microscope Microphot FXA (Nikon, Japan), as well as on the presence of an enzyme specific for Bifidobacteria (fructose-6-phosphate phosphoketolase, EC 4.1.2.22). The lactic acid bacteria were enumerated on MRS medium (BTL, Poland) after undergoing cultivation at 37°C for 72 h in microaerophilic conditions (the double agar layer technique). Their identification was based on appearance of colonies and specific morphology of cells checked under the phase contrast microscope. The quantity of *Escherichia coli *was evaluated after a 24 h incubation at 37°C on McConkey agar (Merck) in an oxidative atmosphere. Spore-forming bacteria were determined after heating of adequate dilutions at 80°C/20 min and cooling. The saccharolytic spore formers were counted after the growth on an SPC medium [19] in anaerobic conditions for 7 days at 37°C, whereas proteolytic spore formers were enumerated in a broth-gelatine medium after 5 days of incubation at 22-25°C in an anaerobic atmosphere [20]. The results were expressed as log cfu number/g digesta. Enzyme activity (α- and β-glucosidase, α- and β-galactosidase, and β-glucuronidase) was measured by the rate of p- or o-nitrophenol release from nitrophenyl glycosides for 10 min [15] and expressed as the μmol of product formed per one min per g of caecal digesta. The SCFA concentration was measured using gas chromatography under the conditions described previously [15]. Briefly, a known amount of fresh caecal digesta was mixed with 0.2 mL of formic acid and stored at -80°C. Afterwards, the sample was diluted with deionized water, centrifuged at 10 000 *g *for 5 min, filtered through a 0.45 μm membrane, and then the supernatant was decanted for injection into a gas chromatograph (Shimadzu GC-14A, Shimadzu Co., Kyoto, Japan, equipped with a glass column, 2.5 m × 2.6 mm, containing 10% SP-1200/1% H_3_PO_4 _on 80/100 Chromosorb W AW; column temperature 110°C; flame ionization detector temperature 180°C; injector temperature 195°C).

The blood serum concentrations of minerals (magnesium, calcium, phosphorous), glucose, triglycerides, and total cholesterol and its HDL fraction, as well as the activity of aspartate aminotransferase (AST) and alanine aminotransferase (ALT) were estimated with reagents from Alpha Diagnostics Ltd. (Warsaw, Poland).

### Statistical analysis

The data were analysed using the STATISTICA software package version 6.0 (Statsoft Corporation, Krakow, Poland). A two-way repeated measures ANOVA was applied to assess the effects of the IN preparation, GFE and the interaction between these dietary factors (IN × GFE). If the analysis revealed a significant interaction or that both dietary factors had a significant influence (*P *≤ 0.05), the differences among the individual groups were then analysed with Duncan's multiple range *post hoc *test (*P *≤ 0.05). The pooled standard error of the mean (SEM) was calculated as the standard deviation from all measurements divided by their square root.

## Results

After four weeks of experimental feeding, the IN and GFE dietary additions did not affect the body weight of rats, their relative liver and kidney mass, serum AST and ALT activity and urea concentration of the rats (Table 2). Dietary GFE treatment significantly increased the tissue and digesta mass and the digesta pH, whereas the digesta dry matter, ammonia and protein concentration were substantially decreased (*P *< 0.001). IN tended to increase the relative caecal tissue mass (*P = *0.09) but significantly reduced the caecal pH value (*P *< 0.001). Moreover, an interaction between IN and GFE was noted with regard to the caecal digesta bulk and its pH value (*P *< 0.05 and *P *< 0.001, respectively). The lowest digesta mass followed the consumption of control (C) and IN diet, whereas the highest digesta mass was in rats on the C + GFE diet (*P *≤ 0.05). The lowest digesta pH value was after the consumption of the IN diet. The digesta pH was significantly higher with the C diet than with the IN diet, whereas the highest digesta pH was in the caecum of rats fed the C + GFE diet (*P *≤ 0.05). The IN + GFE diet had a slightly decreased pH value that was comparable to that observed in rats on the C diet.

**Table 2:** Body weight, mass of liver and kidneys, serum aminotransferase activity and urea concentration, and basic caecal indices of rats consuming control (C) and inulin (IN) diets without or with the addition of grapefruit flavonoid extract (GFE)

Index	Diet				Pooled SEM	ANOVA (*P *value)		
			
	C	IN	C + GFE	IN + GFE		IN	GFE	IN × GFE
Final body weight (g)	257	259	265	265	2.762	NS	NS	NS

Liver								

Mass*	4.44	4.70	4.56	4.58	0.043	NS	NS	NS

ALT (U/l serum)	35.0	36.1	36.3	38.5	1.493	NS	NS	NS

AST (U/l serum)	128	126	127	119	5.123	NS	NS	NS

Kidneys								

Mass*	0.690	0.697	0.683	0.692	0.008	NS	NS	NS

Urea (mg/dl serum)	51.5	49.1	49.0	48.4	1.366	NS	NS	NS

Caecum								

Tissue mass*	0.297	0.327	0.505	0.544	0.022	NS	< 0.001	NS

Digesta mass*	0.895^c^	1.106^c^	2.791^a^	2.242^b^	0.159	NS	< 0.001	< 0.05

Dry matter of digesta (%)	28.0	27.4	18.4	19.7	0.903	NS	< 0.001	NS

Ammonia (mg/g digesta)	0.457	0.400	0.301	0.272	0.018	NS	< 0.001	NS

Protein (mg/g digesta)	0.208	0.214	0.174	0.183	0.004	NS	< 0.001	NS

pH of digesta	7.01^b^	6.65^c^	7.15^a^	7.09^ab^	0.040	< 0.001	< 0.001	< 0.001

ALT, alanine aminotransferase; AST, aspartate aminotransferase.

^a, b, c ^Values not sharing the same superscript letters within a row are different at *P *≤ 0.05.

* g/100 g body weight.

Microbiota and microbial enzyme activity in the caecal digesta of rats are shown in Table 3. Both dietary factors substantially contributed to changes in the bifidobacteria population (*P *< 0.05) but did not affect lactic acid bacteria. The bifidobacteria count was the lowest with the C + GFE diet, slightly and significantly higher with the IN + GFE and C diets, respectively, and highest with the IN diet (*P *≤ 0.05). In addition, the *E. coli *population was considerably decreased after IN supplementation (*P *< 0.001). Dietary GFE reduced significantly the number of saccharolytic spore-forming bacteria (*P *< 0.01), and the number of proteolytic spore-forming bacteria was significantly influenced both by GFE and IN (*P *< 0.001 and *P *< 0.01, respectively). The highest number of anaerobic proteolytic spore formers was observed with the GFE diets. The number of anaerobic proteolytic spore formers was significantly lower with the C diet, and the lowest number of them was observed with the IN diet (*P *≤ 0.05). The activity of all microbial enzymes in the caecal digesta (α- and β-glucosidase, α- and β-galactosidase, and β-glucuronidase, Table 3) was significantly affected by GFE, whereas IN had a substantial influence on α- and β-galactosidase and β-glucuronidase activity; however, a trend of IN towards decreased α-glucosidase activity was also noted (*P = *0.08). Additionally, co-ingestion of IN and GFE led to an interactive effect on α-glucosidase and α- and β-galactosidase activity (*P *< 0.05, *P *< 0.001 and *P *< 0.001, respectively). Specifically, the activity was similar among rats on the C, IN and C + GFE diets and significantly greater in rats on the IN + GFE diet (*P *≤ 0.05). Interestingly, among the aforementioned enzyme activity, α- and β-galactosidase activity in rats on the IN + GFE diet was more than two times and more than five times greater, respectively. The β-glucosidase activity was significantly less with GFE (*P *< 0.001) and was significantly decreased in the caecal digesta of rats consuming IN, C + GFE and IN + GFE diets than in those consuming the C diet (*P *≤ 0.05).

**Table 3:** Microbiota and microbial enzyme activity in the caecal digesta of rats consuming control (C) and inulin (IN) diets without or with the addition of grapefruit flavonoid extract (GFE)

Index	Diet				Pooled SEM	ANOVA (*P *value)		
			
	C	IN	C + GFE	IN + GFE		IN	GFE	IN × GFE
Microbiota*								

Bifidobacteria	9.14^b^	10.35^a^	8.12^c^	8.50^bc^	0.081	< 0.05	< 0.05	NS

Lactic acid bacteria	9.43	9.20	9.17	8.98	0.088	NS	NS	NS

*E. coli*	8.30	6.14	8.42	7.20	0.161	< 0.001	NS	NS

Spore-forming bacteria								

Anaerobic saccharolytic	1.31	1.12	0.92	0.90	0.050	NS	< 0.01	NS

Anaerobic proteolytic	1.19^b^	< 1.0^c^	2.54^a^	1.89^a^	0.113	< 0.01	< 0.001	NS

Enzyme activity^†^								

α-glucosidase	1.14^b^	1.09^b^	1.21^b^	1.99^a^	0.117	NS	< 0.05	< 0.05

β-glucosidase	0.355	0.348	0.191	0.214	0.021	NS	< 0.001	NS

α-galactosidase	0.897^b^	0.586^b^	0.679^b^	2.076^a^	0.155	< 0.05	< 0.05	< 0.001

β-galactosidase	3.29^b^	1.86^b^	2.10^b^	17.14^a^	1.243	< 0.001	< 0.001	< 0.001

β-glucuronidase	1.256^a^	0.687^b^	0.544^b^	0.421^b^	0.090	< 0.05	< 0.01	NS

^a, b, c ^Values not sharing the same superscript letters within a row are different at *P *≤ 0.05.

* log cfu/g digesta.

^† ^μmol/min/g digesta.

The total and all particular SCFA concentrations (acetic, propionic, *iso*-butyric, butyric, *iso*-valeric and valeric acids) in the caecal digesta of rats were significantly influenced by GFE (*P *< 0.01), whereas IN had a substantial influence on the concentration of propionic, butyric and *iso*-valeric acids (*P *< 0.001, *P *< 0.001 and *P *< 0.05, respectively, Table 4). However, valeric acid concentration only trended towards being influenced by the addition of IN (*P *= 0.07). Moreover, an interaction and a trend towards interaction were noted when considering the butyric acid and *iso*-butyric acid concentration (IN × GFE, *P *< 0.001 and *P *= 0.08, respectively). The highest butyric acid concentration was observed with the IN diet, significantly lower with the C diet and the lowest with GFE supplemented diets (*P *≤ 0.05). The total SCFA concentration was almost two times lower in rats on GFE-supplemented diets (*P *< 0.001); the concentration of acetic, *iso*-butyric and valeric acids was also lower in rats consuming these diets (*P *< 0.01). The highest propionic acid concentration was observed with the IN diet, significantly lower with the C and IN + GFE diets and the lowest with the C + GFE diet (*P *≤ 0.05). The *iso*-valeric acid concentration was greater in the caecal digesta of rats consuming the C diet than in rats fed other diets (*P *≤ 0.05). SCFA profile in the caecal digesta of rats were significantly influenced both by GFE and IN (*P *< 0.01). In addition, an interaction was noted when considering the acetic and butyric acid proportion (IN × GFE, *P *< 0.01 and *P *< 0.001, respectively). The highest acetic acid proportion followed the consumption of GFE supplemented diets, significantly lower proportion was observed with the IN diet and the lowest with the C diet. The propionic acid proportion was significantly higher with the IN, C + GFE and IN + GFE compared to the C diet. As in the case of the concentration of butyric acid, its proportion was the lowest in rats on GFE supplemented diets, significantly higher on the C diet and the highest on the IN diet.

**Table 4:** Short-chain fatty acids (SCFA) in the caecal digesta of rats consuming control (C) and inulin (IN) diets without or with the addition of grapefruit flavonoid extract (GFE)

Index	Diet				Pooled SEM	ANOVA (*P *value)		
			
	C	IN	C + GFE	IN + GFE		IN	GFE	IN × GFE
Concentration (μmol/g)								

Acetic acid	31.7	31.9	17.6	21.0	1.359	NS	< 0.001	NS

Propionic acid	3.59^b^	4.81^a^	2.44^c^	3.39^b^	0.185	< 0.001	< 0.001	NS

*Iso*-butyric acid	0.944	0.686	0.569	0.580	0.044	NS	< 0.01	NS

Butyric acid	4.66^b^	8.11^a^	1.42^c^	1.81^c^	0.524	< 0.001	< 0.001	< 0.001

*Iso*-valeric acid	1.12^a^	0.803^b^	0.766^b^	0.706^b^	0.048	< 0.05	< 0.01	NS

Valeric acid	1.400	1.082	0.892	0.821	0.063	NS	< 0.001	NS

Total SCFA	43.4	47.4	23.6	28.3	2.020	NS	< 0.001	NS

Profile (% of total)								

Acetic acid	72·9^a^	67·3^b^	74·2^a^	74·1^a^	0·699	< 0·01	< 0·001	< 0·01

Propionic acid	8·3^b^	10·3^a^	10·4^a^	12·1^a^	0·389	< 0·01	< 0·01	NS

Butyric acid	10·8^b^	17·0^a^	6·0^c^	6·3^c^	0·872	< 0·001	< 0·001	< 0·001

^a, b, c ^Values not sharing the same superscript letters within a row are different at *P *≤ 0.05.

The concentration of glucose, magnesium, calcium and phosphorous did not differ among all diet groups (data not shown). Lipid concentrations in the blood serum of rats are shown in Table 5. Total cholesterol and HDL cholesterol concentrations were comparable among all diet groups, whereas triglyceride concentration was significantly lower in those rats that were fed diets with GFE added.

**Table 5:** Lipid concentrations in the blood serum of rats consuming control (C) and inulin (IN) diets without or with the addition of grapefruit flavonoid extract (GFE)

Index	Diet				Pooled SEM	ANOVA (*P *value)		
			
	C	IN	C + GFE	IN + GFE		IN	GFE	IN × GFE
Total cholesterol (mg/dl)	150	153	165	158	3.655	NS	NS	NS

HDL cholesterol (mg/dl)	33.5	33.8	33.7	34.4	1.051	NS	NS	NS

Triglycerides (mg/dl)	204	197	167	151	9.577	NS	< 0.05	NS

## Discussion

The absorption of flavonoids and their glycosides occurs along the entire gut. Often metabolic processes in the large intestine are predominant and are not fully understood thus far. Among the flavonoids, the bioavailability of the grapefruit flavanones is considered to be relatively high, which seems to be related to their glycosidic moiety. For example, the bioavailability of hesperidin (hesperetin-7-rutinoside) in humans was increased after its enzymatic conversion to hesperetin-7-glucoside, which changed the absorption site from the colon to the small intestine [21]. In another study, naringenin and its glucoside were absorbed across the small intestine of rats, whereas the bioavailability of naringin (naringenin-7-rhamnoglucoside) was significantly lower with the simultaneous compound movement into the caecum [22]. Although there is also a suggestion that naringin may be efficiently absorbed into the bloodstream in its unchanged form, as reported in study on beagles [23], most of the reports suggest that this flavanone and other flavanone glycosides undergo microbial deglycosylation and formation of metabolites, such as phloroglucinol and phenylpropionic acids, in the hindgut [12]. Afterwards, these low-molecular metabolites can be absorbed and might contribute to flavanone bioactivity. In the present study, a 4-week ingestion of a diet containing 0.3% GFE caused a considerable enlargement of the caecal tissue and digesta (Table 2). The amount of GFE used in this study was comparable to the amount of polyphenol extracts usually used in such nutritional experiments. Interestingly, Felgines *et al. *have implied a good adaptation of caecal microbiota to naringin in rats [22]. Our results suggest that the caecal microbiota were not able to sufficiently metabolise the flavonoid glycosides of GFE, and we speculate that it induced additional osmotic pressure that kept fluids within the caecum and led to its enlargement. Nevertheless, because an extract has been used it is difficult to identify specific role of grapefruit phenolics responsible for such observed effects and the influence of other components of GFE cannot be excluded. Interestingly, in our laboratory, high doses of polyphenol extracts (up to 1.2% of the diet) from different sources, including green tea, chokeberry and honeysuckle, have been used so far without a sizeable increase of the caecal tissue and digesta mass [24]. It should be emphasized also that an increase of the caecal digesta mass may cause discomfort to the host. Moreover, such an enlargement of the caecum as in the present study was also observed in a previous experiment performed in our laboratory on rats with lactulose-induced diarrhea [25]. Indeed, in the present experiment, an increased and looser stool output was observed with GFE feeding (data not shown). Notably, the dietary IN beneficially suppressed the increased digesta mass observed after GFE feeding. This finding is especially of interest because IN is well known for increasing faecal biomass and the water content [13] and thus indicates a more complex modulatory effect of IN on the physiology of the large intestine.

Bifidobacteria are known to improve gut health by maintaining a balance between potentially harmful and health-promoting species. Selective stimulation of their growth is one of the most important attributes of IN and other inulin-type fructans [13]. The bifidogenic effect of IN was also observed in our study with an additional decrease in *E. coli *numbers, which some strains can be pathogenic (Table 3). The addition of GFE adversely reduced the number of *Bifidobacterium *spp., but co-ingestion of IN increased the number to a level not significantly different from that observed in rats on the control diet without GFE supplementation. Antibacterial activity of flavonoids has been frequently suggested, but the research is widely conflicting [26]. Although flavonoids are less potent than antibiotics, they are thought to be able to inhibit the nucleic acid synthesis, cytoplasmic membrane function and energy metabolism of bacteria. In our study, dietary GFE affected the growth of only one genus. However, the bacteria investigated are only a small part of the indigenous microbiota, and other caecal indices show more extended microbiota-GFE interactions. Indeed, due to GFE supplementation significantly fewer anaerobic saccharolytic bacteria and more anaerobic proteolytic bacteria were incubated (spore-forming bacteria, Table 3) and it reflects the number of spores present in the caecum. Because spores are formed as response to unfavourable conditions of the environment, it can be concluded that the growth of saccharolytic bacteria was promoted, and the growth of proteolytic bacteria were inhibited, in the caecum of rats fed GFE. Such supposition seems to, at least partly, agree with other indices of caecal function in our study. Evidence of less proteolytic fermentation or less putrefactive processes may be supported by a decreased concentration of their metabolites in the digesta of rats on GFE diets (Tables 2 and 4), such as ammonia, *iso*-butyric, valeric and *iso*-valeric acids [27,28]. The decreased ammonia concentration is considered a positive change because this compound can destroy cells, alter nucleic acid synthesis, induce cancerous cell growth and increase viral infections at concentrations in the lower bowel of usual Western diets [27]. More intensive saccharolytic fermentation indicates microbial glycolytic activity, the activity of α-glucosidase and both galactosidases, which strongly increased after the consumption of the IN + GFE diet (Table 4). However, a several fold increase in β-galactosidase activity is surprisingly high and difficult to explain. Nevertheless, this finding supports the earlier study performed in our laboratory [15]. Interestingly, the β-glucuronidase activity in the caecal digesta of rats fed IN, GFE or both decreased. An increased β-glucuronidase activity has been suggested to be unfavourable due to its ability to liberate toxic or carcinogenic compounds from glucuronide conjugates that are coupled earlier in the liver and excreted into the gut with bile [29].

In the reported experiment, an increased caecal digesta pH was another important disadvantage of GFE consumption (Table 2). Lower pH values are believed to prevent the overgrowth of pH-sensitive pathogenic bacteria and favour the growth of health-promoting species, such as bifidobacteria [30,31]. In our study, the pH values were negatively correlated with the number of bifidobacteria and positively correlated with the SCFA concentration in the caecal digesta. Interestingly, the co-ingestion of IN slightly decreased the pH to a level not significantly different from that observed with the control diet without GFE supplementation; yet, the digesta was acidified only in rats consuming IN as a single dietary supplement. The total SCFA concentration was almost two times lower with diets with GFE supplementation (Table 4). There is mounting evidence that SCFA play a key role in colonic health as an energy source for colonocytes, and some may be an important factor for the prevention and management of certain diseases, such as gastrointestinal disorders, cancer or CVD [31]. In our study, the acetic acid concentration in the caecal digesta decreased significantly after GFE supplementation. This finding could be considered a positive change because it is believed that after absorption, acetate stimulates cholesterol synthesis in the liver; however, GFE consumption simultaneously decreased the propionate concentration, which is suggested to be an inhibitor of hepatic cholesterol synthesis [31]. A lack of a cholesterol-lowering effect with GFE in our study might be a consequence of that interaction. Nevertheless, GFE and IN in the diet together significantly increased the propionic acid concentration, which was comparable to that observed in rats on the control diet without additives. The highest concentration of propionic acid and butyric acid was after ingesting IN as a single supplement. This finding is consistent with the well-known ability of IN to stimulate bacterial propionate and butyrate production in the large intestine [13]. In the present experiment, a several fold decrease in the butyric acid concentration was also observed in rats on GFE diets. Butyrate is thought to be the preferred energetic substrate for colonocytes, and it appears to promote a normal phenotype of these cells [30,31].

The serum cholesterol concentration was similar among all dietary groups in our study; however, the serum triglyceride concentration decreased significantly in rats consuming GFE (Table 5). This finding supports that of the study by Cho *et al. *performed on rats consuming a high-sucrose diet and dietary levels of naringenin at concentrations equivalent to 1-4 cups of grapefruit juice, which significantly lowered blood triglyceride concentrations but had an ambiguous effect on blood cholesterol [32]. The authors suggest that the lipid-lowering effects may result from the upregulation of hepatic peroxisome proliferator-activated receptor alpha and its target genes by naringenin. Surprisingly, in another study of rats fed a cholesterol-containing diet, a solution of hesperidin or naringin given once daily by gavage significantly hindered an increase in the plasma total cholesterol and LDL but had no influence on plasma triglycerides after 30 days [7]. Additionally, human trials are inconsistent in terms of the lipid-lowering effects of grapefruits and their flavonoids. For example, fresh red grapefruit positively influenced all serum lipid levels, especially serum triglycerides, in hyperlipidaemic patients after coronary bypass surgery [33]. However, in a randomised, placebo-controlled, parallel trial, pure hesperidin and naringin did not affect serum lipids in moderately hypercholesterolaemic men and women [34]. Other grapefruit components may also play a role in the lipid-lowering effects. In the present study, contrary to the expectations, dietary IN did not affect the serum lipids despite an increased concentration of propionic acid in the caecal digesta. A meta-analysis of randomised controlled trials shows that the triglyceride-lowering effect of inulin-type fructans is well proven [35]. In the reported study, the serum mineral concentrations were not affected by IN or GFE. This finding does not support the literature that reports a potent improved mineral metabolism with IN and grapefruit flavanones [8,11,14]. Perhaps the period of low-grade metabolic acidosis from the Western-type diet was too short in our study to disturb the mineral homeostasis of rats.

## Conclusion

Inulin does not provide any additional benefit to the blood lipid profile caused by dietary application of the grapefruit flavonoid extract and it does not counteract clearly detrimental effects of the extract in the hindgut. Adding grapefruit extract to the diet must be performed with caution due to possible adverse hindgut responses with overdoses.

## Abbreviations

ALT: alanine aminotransferase; AST: aspartate aminotransferase; C: control; CVD: cardiovascular disease; GFE: grapefruit flavonoid extract; IN: inulin; SCFA: short-chain fatty acids

## Competing interests

The authors declare that they have no competing interests.

## Authors' contributions

AJ, ZZ and JJ were involved in the conception of the study; JJ, AJ and KK conducted the research; AJ prepared the manuscript. A critical revision of the text was completed by all authors. All authors read and approved the final manuscript.
